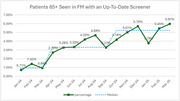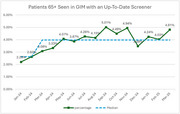# Cognition Counts: Improving Cognitive Impairment Screening Rates for Adults 65+ in Primary Care

**DOI:** 10.1002/alz70861_107973

**Published:** 2025-12-23

**Authors:** Natalie Sanfratello, Lisa Caruso, Susan Phillips, Heather Ross, David Yuh, Gertha Dabady, Sanford H. Auerbach, Alison Li, Hollis Day

**Affiliations:** ^1^ Boston University Chobanian & Avedisian School of Medicine, Boston, MA USA; ^2^ Boston Medical Center, Boston, MA USA

## Abstract

**Background:**

Cognitive screening in primary care is critical for timely diagnosis /intervention for dementia, but there are barriers to implementation. In ambulatory clinics in a safety net hospital, patients face barriers related to access, language, and socioeconomic challenges while clinicians face barriers of limited time, availability of tools, knowledge of which screening tools to use, and what to do if a screen is positive.

**Methods:**

From March 2024 to March 2025, QI teams in family medicine (FM) and general internal medicine (GIM) along with geriatricians and neurologists at Boston Medical Center (BMC) and QI managers from the Center for Continuing Education at Boston University Chobanian & Avedisian School of Medicine (BU CCE) executed a QI project leveraging the Model for Improvement focused on increasing cognitive screening rates in primary care. The aim was to increase the percentage of patients aged ≥ 65 with a cognitive screening score (within the last 12 months) from 0.92% in FM and 2.43% in GIM to 6% in one year. Cognitive screening rates were quantified based on the proportion of completed RUDAS, MMSE, MiniCog, or MoCA questionnaires in patients aged ≥ 65 without a dementia diagnosis attending either primary care clinic. Based on gap analyses findings we implemented multiple interventions via PDSA cycles including updates to the EMR, implementation of nurse‐ and pharmacist‐driven screening, improved guidance for referrals, and educational interventions.

**Results:**

In the run chart, FM saw two shifts, a peak of 5.97% in March 2025. GIM saw one shift in the run chart, a peak of 5.01% in September 2024 and ended with 4.81% in March 2025.

**Conclusions:**

Regular cognitive screening in these primary care settings remains low, but statistically significant improvements were seen based on run chart rules. Including more members of the interprofessional care team made screening easier and more sustainable. These improvements were realized in the absence of CMS Annual Wellness visits and in a setting that treats a high percentage of at‐risk patients.